# Wound of the Abdomen; Delirium Tremens

**Published:** 1844-10-05

**Authors:** 


### WOUND OF THE ABDOMEN; DELIRIUM TREMENS.

Mr. Tennent narrates, in the London and Edinburg Medical Journal, a case of penetrating wound of the abdomen inflicted on a drunkard, who was going on well for a few days, when delirium tremens set in, requiring the free exhibition of opium and ammonia. The case ultimately did well.

